# Repair of long bone defects of large size using a tissue-engineered periosteum in a rabbit model

**DOI:** 10.1007/s10856-021-06579-7

**Published:** 2021-08-21

**Authors:** Lin Zhao, Junli Zhao, Zhenhe Tuo, Guangtie Ren

**Affiliations:** 1 Orthopedic Department of Guangming Traditional Chinese Medicine Hospital of Pudong New Area, Shanghai, China; 2 Department of Nephrology, Shanghai University of Medicine & Health Sciences affiliated Zhoupu Hospital, Shanghai, China; 3 Orthopaedic Department of Xianyang Central Hospital, Shaanxi Province, People’s Republic of China; 4 Orthopaedic Department of Hanzhong Central Hospital, Shaanxi Province, People’s Republic of China

## Abstract

Tissue engineering is a promising approach for bone regeneration. In this study, we aimed to investigate whether tissue engineered periosteum (TEP), which was fabricated by combining osteogenically-induced mesenchymal stem cells (MSCs) with porcine small intestinal submucosa (SIS), could restore long bone defects of large size in rabbits. Twenty-four adult New Zealand white rabbits (NZWRs) were used in the experiments. Long bone defects of large size (30 mm-50 mm; average, 40 mm) were established on both sides of NZWRs’ radii. The defects were treated with TEP (Group A), allogeneic deproteinized bone (DPB, Group B), TEP combined with DPB (Group C), and pure SIS (Group D). The healing outcome was evaluated by radiography and histological examination at 4, 8, and 12 weeks post-treatment. The radiographical findings showed that bone defects of large size were all repaired in Groups A, B and C within 12 weeks, whereas Group D (pure SIS group) failed to result in defect healing at 4, 8, and 12 weeks. Although there was some new bone regeneration connecting the allografts and bone ends, as observed under radiographical and histological observations, bone defects of large sizes were restored primarily by structurally allografted DPB within 12 weeks. The TEP groups (Groups A and C) showed partial or total bone regeneration upon histological inspection. Based on 12-week histological examinations, significantly more bone was formed in Group A than Group C (*P* < 0.05), and both groups formed significantly more bone than in Groups B and D. The results indicated that long bone defects of a large size could be restored by TEP or TEP combined with the DPB scaffold, and such materials provide an alternative approach to resolving pathological bone defects in clinical settings.

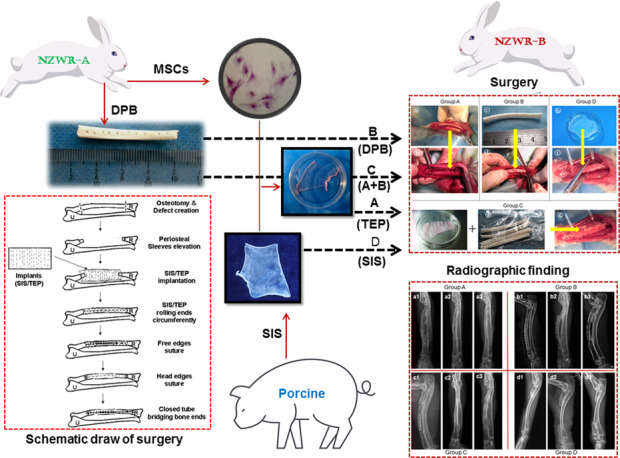

## Introduction

Segmental bone defects due to trauma, cancer, or congenital deformity are major clinical challenges [1, 2]. A promising approach for bone regeneration is bone tissue engineering (BTE), which is based on in vitro manipulation of seed cells, growth factors, and scaffolds [3, 4]. While much effort has been devoted to BTE, very little of such research has been translated to the clinic [1, 4]. First, the process of tissue engineering bone construction is highly complex and its efficiency remains relatively low [5]. Second, large-bone regeneration has failed previously due to the lack of the vascularization within the BTE constructs and slow penetration of the host vasculature; thus, resulting in poor implant survival and integration [6, 7]. In the absence of capillary networks within the 3D implants, the engineered tissues can only have a maximum thickness of 150–200 mm; dimensions larger than this threshold may result in a lack of oxygen inside the biomaterials [8].

In clinical situations, bone defects requiring surgical treatment can encompass any size and shape of tumor or other bone disease. The size of newly formed bone should be suitable for the patient’s specific anatomical pathology, as well as for routine clinical application [9, 10]. Traditional BTE treatments are not likely to restore pathological defects in their original size and shape. Therefore, intensive efforts should be made to seek alternative approaches.

An alternative strategy to traditional BTE treatments includes using the body as a bioreactor to cultivate the traditional BTE construct, and leverage the body’s own regenerative capacity to regenerate new bone tissue and vessels [1]. Thus, a hybrid of the in vivo bioreactor (IVB) principle and a tissue-engineered osteogenic construct may be a promising approach to repair bones defects of all sizes.

The periosteum plays a key role in both bone development and bone healing via endogenous repair mechanisms [9, 10]. Studies have reported that bone defects could be effectively repaired using osteogenic cell sheets or periosteal implantations [11–15]. However, cell sheets failed to maintain specific forms without scaffold support because of their weak strength and tenacity. Therefore, the cell sheet strategy is limited and cannot be used directly to repair defects of large segmental bone [5]. However, it would provide great promise to fabricate a biomimetic periosteum substitute that could fit any size or shape for use in large-bone-defect repair [16].

In our previous studies, we fabricated a biomimetic construct of tissue-engineered periosteum (TEP) by combining rabbit osteogenically-induced MSCs with porcine small intestinal submucosa (SIS) [17, 18]. Such constructs efficiently restored critical sized bone defects (CSD) in an allogeneic rabbit model by suturing TEP sheets into a relatively closed chamber, using the principles of IVB [19, 20].

In order to simulate the clinical repair of long bone defects of any size needed for individual patients. Here, we investigated whether the self-assembling TEP could restore large segmental bone defects of any size.

## Methods

### Experimental animals

All animal procedures were carried out in strict accordance with the regulations governing medical animal experiments. Twenty-four adult New Zealand White rabbits (NZWRs, male and female, 2–3-months-old, weighting 2.5–3.5 kg) and 6 neonatal NZWRs (male and female, 7–10 days old, weighing 0.2–0.4 kg) were provided by the Animal Experiment Center of the Lanzhou Institute of Biological Products (Lanzhou, Gansu province, China). Among of these 30 rabbits, 24 adults were used for in vivo study because we would like to create a large size bone defect and reparative model. While 6 neonatal rabbits were sacrificed under anesthesia for cell isolation because red bone marrow is richer in neonatal rabbits than that of adults. All of the experimental procedures involving animals were conducted in accordance with the Institutional Animal Care Guidelines of Lanzhou University, China, and were approved by the Institutional Animal Care and Use Committee of Lanzhou University, Gansu Province, China (IACUC no. 2016016).

### Seeding cells

Six neonatal rabbits (male and female, 7–10 days old, weighting 0.2–0.4 kg) were sacrificed with an overdose of pentobarbital (20%) injected in an ear vein for cell isolation. Bone marrow was aspirated from the femurs and tibias under aseptic conditions. MSCs were isolated by density gradient centrifugation using Percoll (Invitrogen, Carlsbad, CA, USA). The homogeneously suspended cells were counted on a hemocytometer and seeded in 50 mL culture flasks (1.0 × 10^6^ nucleated viable cells per flask). The culture medium was Dulbecco’s Modified Eagle’s Medium (DMEM, Invitrogen) supplemented with 10% fetal bovine serum (FBS, Sijiqing Biological Engineering Materials Co., Ltd. Hangzhou, China), penicillin (100 U/mL), streptomycin (100 U/mL), and 3% L-Glutamine (Sigma-Aldrich, St. Louis, MO, USA). The cells were incubated at 37 °C in a humidified incubator with 5% CO_2_ for 5 days before changing the medium. Cells were then washed with PBS and the non-adherent cells were removed. The medium was replaced twice per week. The cells were subcultured by digesting with a coenzyme solution of 0.125% trypsin (Gibco, Carlsbad, CA, USA) and 0.02% ethylenediaminetetraacetic acid (EDTA, Invitrogen) when the cultures reached 90% confluence.

The third passage of MSCs was selected for osteogenic differentiation. The MSCs were induced for 3 weeks in DMEM (Invitrogen) containing 10% FBS (Sijiqing) supplemented with 50 mg/L sodium ascorbate, 10 mmol/L sodium β-glycerol phosphate, and 10 nm/L dexamethasone (all from Sigma–Aldrich) at 37 °C in a humidified chamber with 5% CO_2_. Osteogenic differentiation of MSCs was assessed using the Gomori method to detect alkaline phosphatase activities (ALP) and Alizarin Red S (Najing Jiancheng Biotechnology Co. Ltd, China) staining was used to detect mineralized nodules.

### Preparation of the SIS scaffold

A segment of fresh porcine jejunum was harvested from healthy pigs (about 100 kg at 6 months old, Lanzhou Slaughter Factory, Gansu Province, China) within 4 h of sacrifice. Porcine small intestinal submucosa (SIS) was prepared by a multi-step procedure, including mechanical disassociation, degreasing, enzymatic digestion, detergent treatment, lyophilization, and sterilization to form a decellular sheet scaffold [21]. SIS was obtained by mechanical removal of the tunica serosa and tunica muscularis, and cleaned by washing continuously with Phosphate buffered solution (PBS). Next, the submucosa was submerged in a solution containing methanol and chloroform (1:1, v/v) in a fume hood for 12 h, and rinsed with the deionized water to remove the organic solvents. The membrane was incubated in 0.05% trypsin/0.05% EDTA (Invitrogen) at 37 °C for 12 h and then rinsed with PBS to remove the trypsin. Subsequently, the membrane was further treated with 0.5% sodium dodecyl sulfate (SDS) in 0.9% sodium chloride by continuous shaking for 4 h. Then, the detergent was removed by thoroughly rinsing with PBS. Finally, the submucosa was soaked in 0.1% peroxyacetic acid and 20% ethanol for 30 min, and rinsed with PBS. All samples were freeze-dried at −70 °C, sealed in hermetic packages, and sterilized with Co_60_ gamma irradiation (25 kGy) for 30 min (Lanzhou Radiation Centre, Lanzhou, China).

### TEP fabrication

SIS sheets were spread onto the bottom of culture dishes, and then disinfected under ultraviolet light for >1 h. Pure FBS was added to pre-wet SIS sheets for more than 12 h. Then, 500 μL of osteogenically-induced MSCs at a density of 1 × 10^6 ^cells/mL were added onto the surface of the pre-wetted SIS, and incubated for 4 h to allow for cell attachment. Additional DMEM with 10% FBS was supplemented onto the cells, and the culture was incubated at 37 °C in 5% CO_2_ and 95% humidity for 10–15 days to form bioactive TEP. The medium was changed every 2–3 days.

Pure FBS pre-wetting SIS sheets was aimed to enhance cell attachment for TEP fabrication, and 10% FBS in DMEM was a normal component of nourishment for MSCs culture. FBS and DMEM were cleaned by washing continuously with PBS upon implanting,in case of potential immunoreaction aroused by heterogenic protein.

### Preparation of the DPB scaffold

Segmental radial shafts of NZWRs were harvested using surgical procedures and used to develop the defect model. Shafts were deproteinized and made into whole blocks after the attached soft tissue was removed. Vertical holes (0.5 mm in diameter) were drilled into each block following deproteinization. The bone blocks were then treated sequentially with H_2_O_2_, NaN_3_, NaOH, protease, a methanol/chloroform mixture, ether, ethane diamine, and absolute alcohol to produce DPBs [22]. The samples were then dried at 50 °C in a drying oven, sealed in hermetic packages, and then sterilized using Co_60_ gamma irradiation (25–35 KGY).

### Scanning electron microscopy (SEM)

SEM analysis was carried out to characterize the morphology of membranous implants. Either SIS or TEP membranes were fixed in 2% glutaraldehyde and then each specimen was subjected to sequential dehydration in 30, 50, 70, 90, and 100% ethanol for 15 min each. The final incubation in 100% ethanol was performed three times after replacing with fresh ethanol each time. Following this, specimens were subjected to critical point drying and gold sputter coating. The surface ultrastructure and morphology of the TEP and SIS were then visualized using SEM (JSW-680LA, Japan).

### Animal surgery

In strict accordance with the regulations for medical animal experiments, 24 adult NZWRs (male and female) were anesthetized intraperitoneally by injection of 3% pentobarbital solution (40 mg/kg body weight). Rabbits were then placed in the prone position. The upper limbs were shaved and aseptically prepared for surgery. A 5–6 cm longitudinal skin incision was made in the bilateral forearm, and the soft tissue was then separated to expose radial shaft. The segmental diaphysis of 3.5–5 cm (average, 4 cm) along with the attached periosteum was removed using an oscillating saw to establish a long bone model of large-sized defects.

**Fig. 1:**
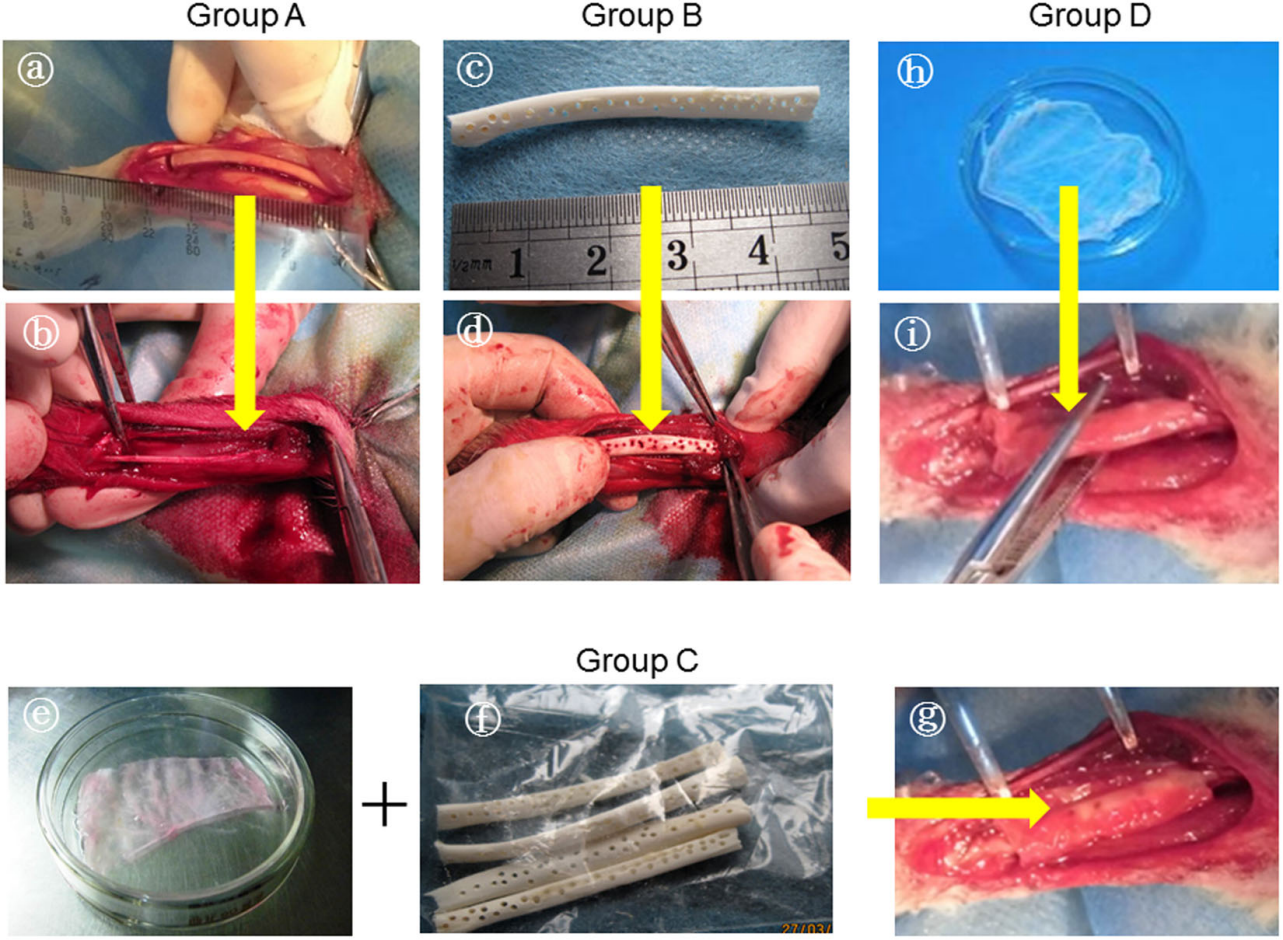
Bone defects of any size were established by osteotomy (**a**). Bone defects were bridged by TEP in Group A (**b**), by an allogeneic DPB block in group B (**c** and **d**), by TEP-wrapped DPB in Group C (**e**, **f** and **g**), and by pure SIS in Group D (**h** and **i**)

Then, 48 large bone defects in 24 animals were randomly divided into four groups. Defects were then treated with TEP (Group A, *n* = 12), DPB (Group B, *n* = 12), a hybrid of TEP and DPB (Group B, *n* = 12), and pure SIS (Group D, *n* = 12). TEP or SIS (Group A or D) was tailored to fit the size of defect, then rolled circumferentially around both of the bone ends of defects. The free edges of the sheets were sutured together, edge-by-edge with a 7–0 atraumatic suture, while the end edges of sheets around the bone ends were sutured to the periosteal sleeves, which were then elevated from the bone ends following osteotomy. The type of suture rendered the membranous implant into a tubular compartment to bridge the defect gap (Fig. 1). For DPB implantation treatments (Group B), both ends of the DPBs were properly tailored to fit the length of each defect area. They were then applied to the defect area and adhered tightly between the bone ends. Then, multiple threads (nylon suture 10) were passed through the holes of the DPBs, which were drilled during the deproteinization process (described above), and the holes in the bone ends that were drilled immediately prior to DPB implantation. The threads were tied tightly to fix the DPBs or TEP-covered DPBs to the bone ends. For DPB + TEP grafting (Group C), the DPB implanting and fixing procedure was similar to that performed in Group B, with the addition of a TEP-wrapping step around the DPB (on its axis), and a suturing step similar to that performed in Group A. Figure 1 shows the surgery performed in the long bone model of defects of any size.

The incisions were then closed layer-by-layer using a 1-0 nylon suture. The forelimbs of rabbits were immobilized for 4 weeks using plaster casts. The rabbits received 400,000 units of penicillin per day, postoperatively for 3 days to prevent infection.

### Radiographic analysis

Bone healing was monitored by radiographs (DR 3000 Dryview8900, Koda, Japan) at 4, 8, and 12 weeks post-operation. The standardized anteroposterior and lateral views of the radius were performed by X-ray radiography at 4, 8, and 12 weeks. The area of new bone formation was quantified as a percentage of the total area of the defect using radiographs at 12 weeks. Each radiograph was judged by three independent examiners that were blinded from all of the groups. The Lane-Sandhu scoring system was applied to evaluate radiographic outcomes (Table 1) [23, 24]. Radiographic scores were compared between the groups.

**Table 1:** Lane–sandhu radiographic scoring standard

Category	Standard	Scores
Bone formation	No evidence of bone formation	0
	Bone formation occupying 25% of defect	1
	Bone formation occupying 50% of defect	2
	Bone formation occupying 75% of defect	3
	Full gap bone formation	4
Fracture line	Clear	0
	Relatively clear	1
	Partial fracture line	2
	Basically vanished	3
	Completely vanished	4
Bone remodeling	No evidence of remodeling	0
	Remodeling of medullary canal	2
	Full remodeling of cortex	4

### Histological examination

Animals were sacrificed under anesthesia with an overdose of pentobarbital (20%) injected in an ear vein at 4, 8, and 12 weeks post-operation to harvest the specimen for histological examination.

Samples of the defect area were excised for histological examination. Specimens were transected, and fixed with 4% paraformaldehyde in a 0.1 M phosphate buffer (pH 7.4) for 3 days. Following fixation, specimens were decalcified with 10% EDTA (Invitrogen) solution for 4 weeks at 4 °C. The tissues were cut into 7 mm sections and stained with hematoxylin and eosin (HE) to evaluate the newly formed bone and residual scaffold using light microscopy. For morphometric analysis, the Lane-Sandhu scoring system (Table 2) was applied to evaluate histological outcomes [23, 24]. Histological scores were compared between the groups.

**Table 2:** Lane–sandhu histological score standard

Category	Standard	Scores
Union	No sign of union	0
	Fibrous union	1
	Osteochondral union	2
	Bone union	3
	Complete reorganization	4
Spongiosa	No sign of cellular activity	0
	Early bone formation	1
	Active new bone formation	2
	Reorganized spongiosa formation	3
	Complete reorganized spongiosa	4
Cortex	Absence of cortex	0
	Early detection	1
	Initiation of formation	2
	Reorganization in majority	3
	Complete organization	4

### Statistical analysis

Statistical analysis was performed using SPSS 15.0 software. All quantitative data are expressed as the mean ± standard deviation (M ± SD). Statistical comparison was performed by one-way analysis of variance (ANOVA). Statistical significance was considered at a probability <0.05.

## Results

### Seeding cells and implants

**Fig. 2:**
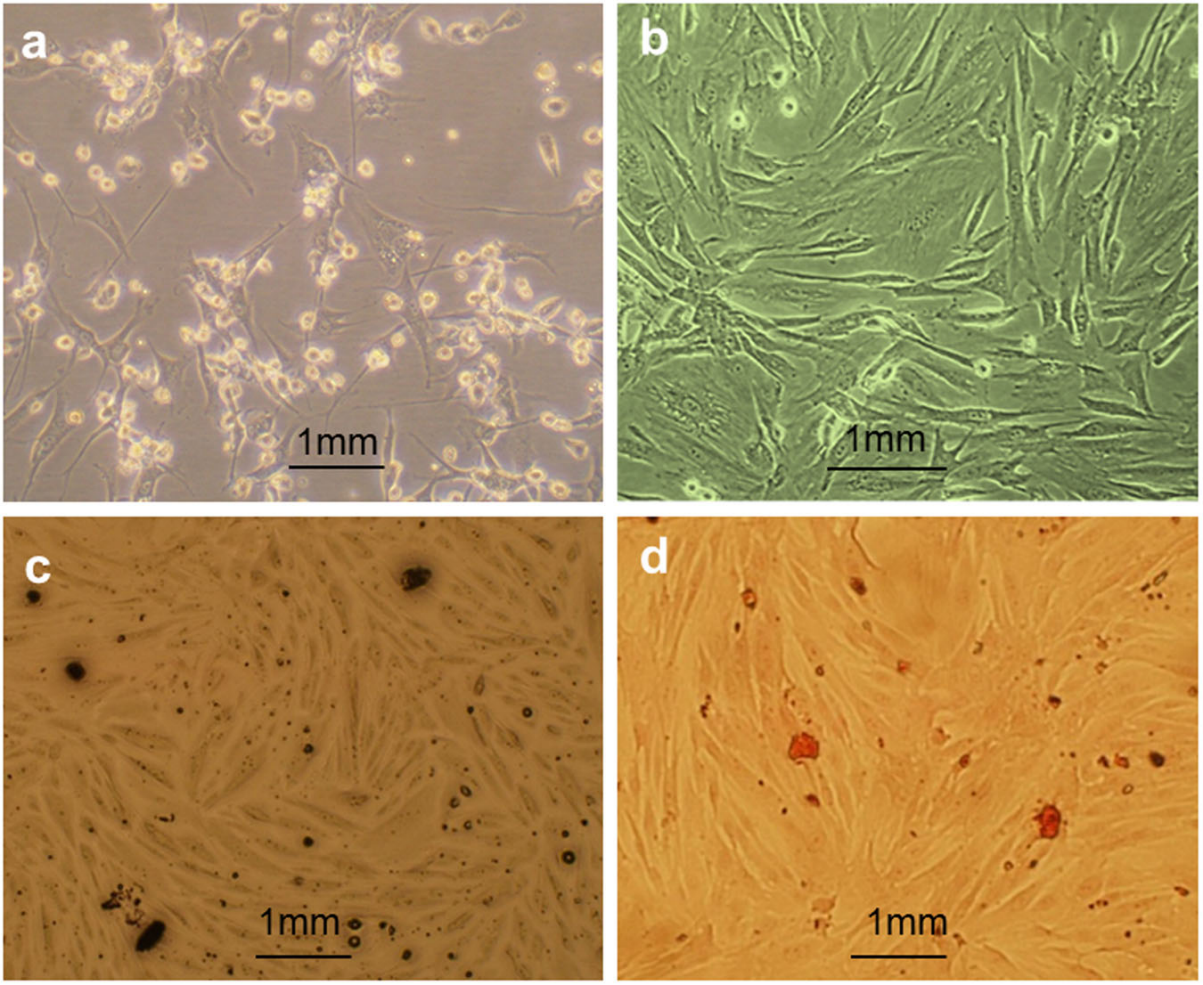
Morphological investigation of the primary MSCs (**a**), osteogenically induced MSCs (**b**), AKP staining (**c**) and Alizarin Red staining (**d**) under light microscopy

Under light microscopy, the homogenated MSCs exhibited a typical fusiform-shaped and fibroblast-like appearance (Fig. 2a). After being osteogenically induced for 3 weeks, the morphology of the cells changed to a multi-angle appearance with several processes (Fig. 2b). The osteogenic differentiation was further identified by AKP staining (Fig. 2c), and the formation of mineralized nodules determined by Alizarin Red staining (Fig. 2d).

**Fig. 3:**
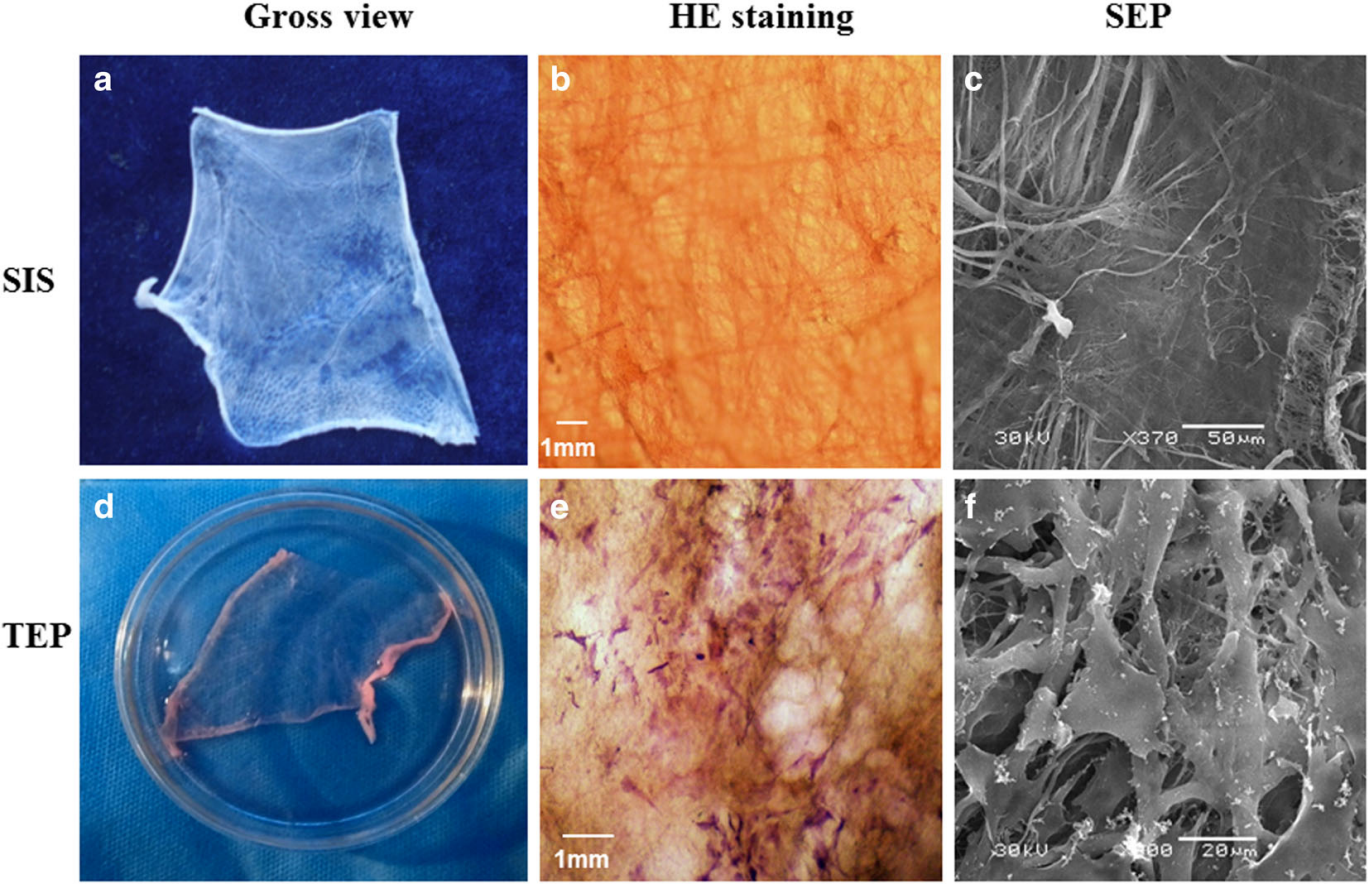
SIS evaluated under macroscopy (**a**), HE staining (**b**) and SEM (**c**). TEP inspected under macroscopy (**d**), HE staining (**e**) and SEM (**f**)

The homemade SIS was a white elastic sheet with a thickness of 100 ± 20 μm, and a texture similar to the natural periosteum, as determined by macroscopic assessments (Fig. 3a). SIS revealed a network structure formed by woven fibers under light microscopy (Fig. 3b) and SEM (Fig. 3c).

Based on gross views, the TEP implants preserved the flexible and membranous nature of the SIS (Fig. 3d). HE staining indicated that there were abundant seeding cells attached to the TEP (Fig. 3e). Under SEM, the TEP showed a non-woven fibrous structure with cells attached (Fig. 3f).

### Physical examination of animals

All animals recovered within 2 h after surgery and the wounds were cured within 10 days. In ~2 weeks, animals were able to move under caster protection. All animals remained in normal health throughout the course of the experiment.

### Macroscopic view

**Fig. 4:**
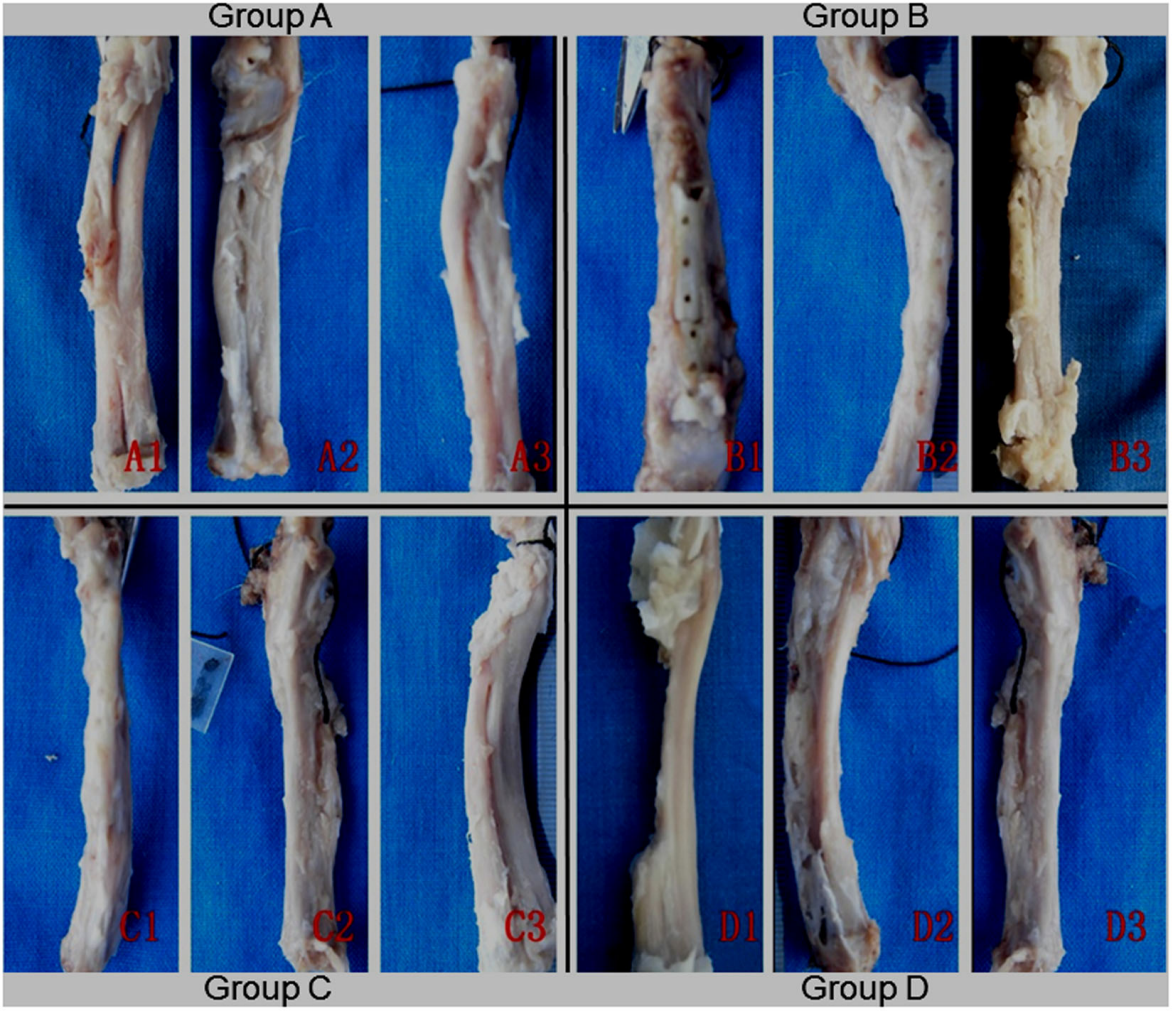
Macroscopic view of bone defects repair in Group A at 4 weeks (**A1**), 8 weeks (**A2**) and 12 weeks (**A3**); in Group B at 4 weeks (**B1**), 8 weeks (**B2**) and 12 weeks (**B3**); in Group C at 4 weeks (**C1**), 8 weeks (**C2**) and 12 weeks (**C3**); and in Group D at 4 weeks (**D1**), 8 weeks (**D2**) and 12 weeks (**D3**)

All sizes of bone defects exhibited complete union in Groups A and C, and were mainly connected by DPB in group B. Group D defects remained nonunion (Fig. 4).

### Radiographic examination

**Fig. 5:**
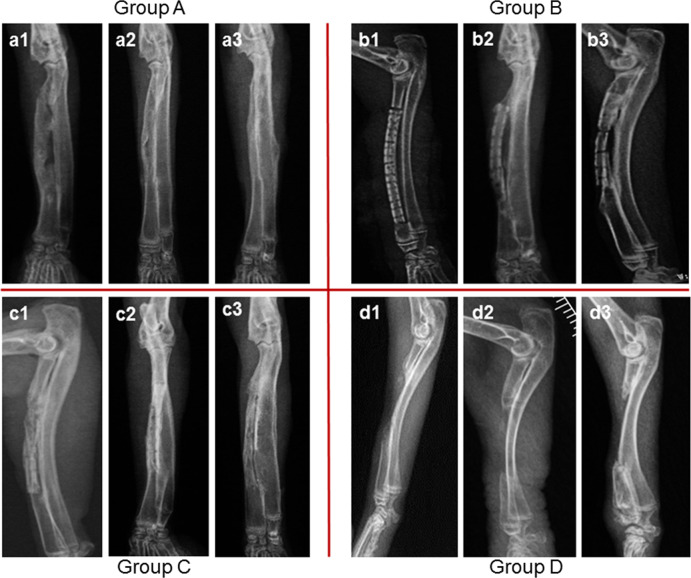
Radiological view of bone defect repair in Group A at 4 weeks (**a1**), 8 weeks (**a2**) and 12 weeks (**a3**); in Group B at 4 weeks (**b1**), 8 weeks (**b2**) and 12 weeks (**b3**); in Group C at 4 weeks (**c1**), 8 weeks (**c2**) and 12 weeks (**c3**); and in Group D at 4 weeks (**d1**), 8 weeks (**d2**) and 12 weeks (**d3**)

The progress of bone defect repair was analyzed by radiography with parallel comparisons between all groups. In Group A, a low-density callus formed at the middle of the defects, as well as in the adjacent zone of the implants and bone ends at 4 weeks. New bone formation was increased, cancellous bone was formed, and a complete bony union was formed at 8 weeks. Bone remolding was achieved with mature cortical bone and a medullary canal at 12 weeks. In Group B, holes in the DPB and the gap between the DPB and bone ends were radiographically non opaque at 4 weeks, which indicated that no obvious bone formation occurred at any sites within the defect area. At 8 weeks, the holes in the DPB remained radiographically non opaque, but the adjacent gaps between the DPB and bone ends exhibited new bone filling the adjacent zone. Meanwhile, the decreased density of the DPB revealed degradation at this stage. At 12 weeks, holes at the middle sites remained radiographically non opaque, but the holes at the DPB ends and adjacent gaps were almost radiographically invisible. Both ends of the DPB seemed to be partially substituted by new bone. Large defects in this group were restored by partial DPB and partial new bone at this stage. In Group C, holes in the DPB, and gaps between the DPB and bone ends, were filled by new bone at this early stage. At 8 weeks, holes and gaps were invisible, which indicated that the DPB was partially replaced by new bone. At 12 weeks, the DPBs were almost invisible and the defect areas were replaced by new bone with immature medullary canals and nonuniform cortices. In Group D, defect areas were radiographically non opaque at 4, 8, and 12 weeks. In addition, there were only a few of spurs forming around of bone ends in this group at 8 and 12 weeks (Fig. 5).

**Fig. 6:**
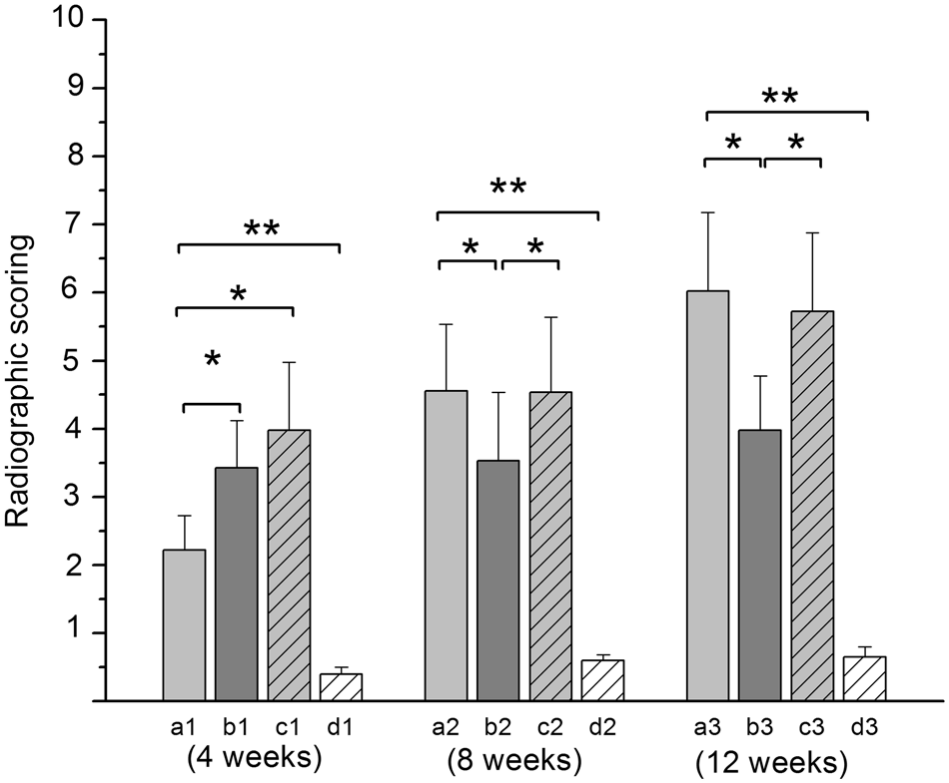
Histograms of radiological scores between each group at 4, 8 and 12 weeks. The letters **a1**, **a2** and **a3** represent Group A at 4, 8 and 12 weeks, respectively. In the same way, **b1**, **b2**, **b3**, **c1**, **c2**, **c3** and **d1**, **d2**, and **d3** represent Groups B, C and Group D at 4, 8, and 12 weeks, respectively. Values are expressed as the mean ± S.D.; *n* = 12 per group. “*” and “**” represent significant differences between the different groups at the same time (*P < 0.05 and **P < 0.001)

Semiquantitative comparisons of radiological scores showed that Group D had the lowest score at 4, 8, and 12 weeks (*P* < 0.001). Radiological scores in Group B and Group C were not different (*P* > 0.05), but were significantly higher than Group A and Group D (*P* < 0.05) at 4 weeks. At 8 weeks, radiological scores in Group A had increased to that of Group C (*P* > 0.05), whereas they were significantly higher than that of Group B and Group D (*P* < 0.05). At 12 weeks, radiological scores in Group A and Group C increased equivalent amounts (*P* > 0.05), and were significantly higher than that of Group B and Group D (*P* < 0.05) (Fig. 6).

### Histological analysis

**Fig. 7:**
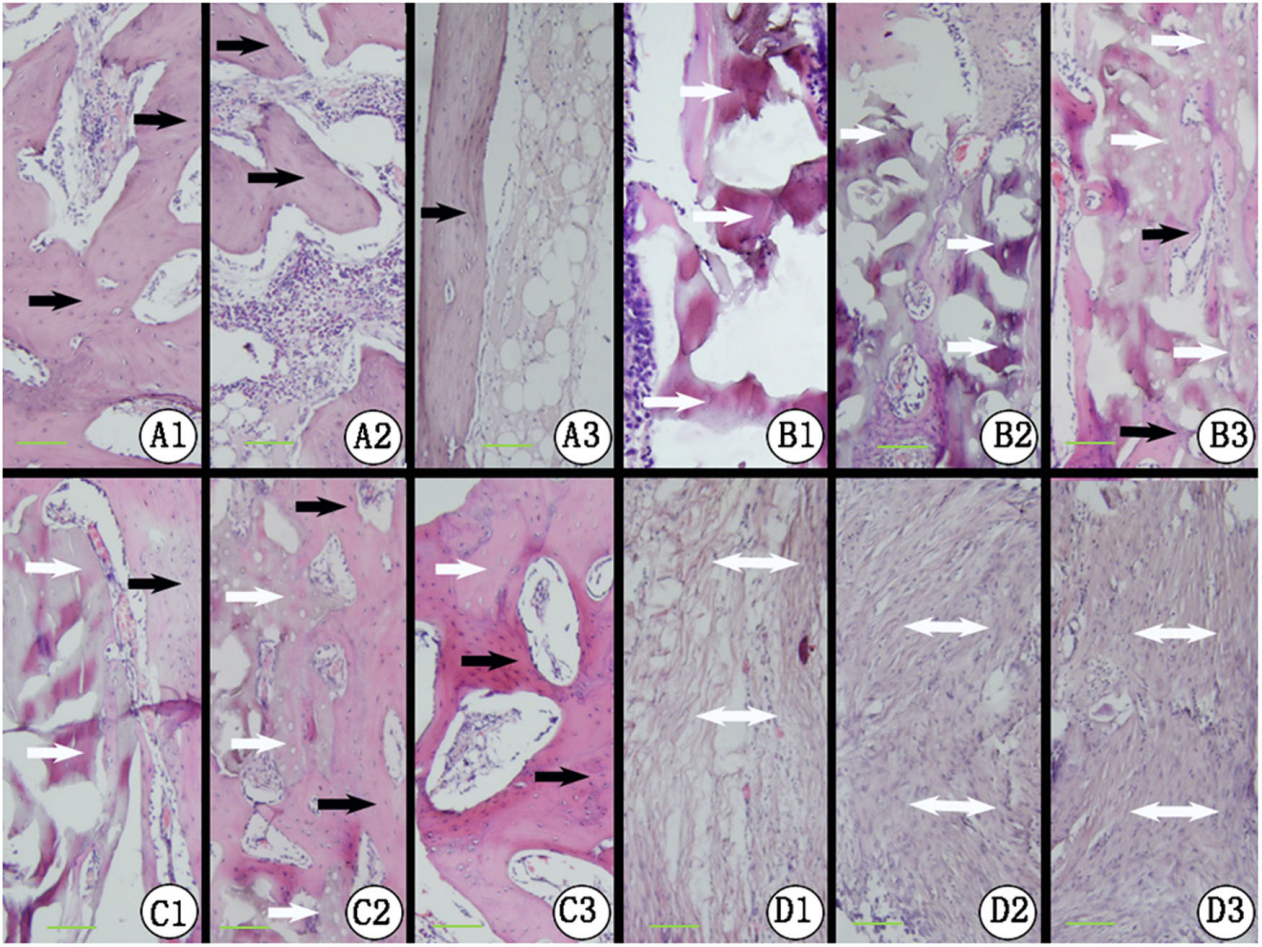
Histological inspection of HE staining in Group A at 4 weeks (**A1**), 8 weeks (**A2**) and 12 weeks (**A3**); in Group B at 4 weeks (**B1**), 8 weeks (**B2**), and 12 weeks (**B3**); in Group C at 4 weeks (**C1**), 8 weeks (**C2**) and 12 weeks (**C3**); and in Group D at 4 weeks (**D1**), 8 weeks (**D2**), and 12 weeks (**D3**). The black arrow represents new bone, the white arrow represents DPB remnants, and the scale bar represents 1 mm

Under light microscopy, the defect site of the radius in Group D revealed mainly scar tissue with limited mineralized tissue at the bone ends at 4, 8, and 12 weeks post-implantation, which was confirmed by macroscopic specimen at 12 weeks. In Group B, new bone formation was observed and accompanied by DPB degradation from weeks 4 to 12. However, TEP groups (Group A and C) exhibited increased new bone formation from 4 weeks to 12 weeks post-operation. Defects in Group A were evenly filled with immature calluses accompanied by some degradation of the TEP at the middle of the defects and the adjacent area after 4 weeks. At 8 weeks, cancellous bone was evenly formed and accompanied with irregular vessels or medullary cavities, while the TEP degradation products were not observed at this stage. Mature cortical bone tissue and medullary cavities were remodeled to achieve a mature long-bone structure at 12 weeks. In Group C, new bone penetrated evenly into the pores of the DBP, accompanied by some degradation of the DPB and TEP at the proximal, distal, and middle sites of the defects after 4 weeks. At 8 weeks, a portion of the DPB scaffold was substituted by new bone tissue, whereas the DPB was still discernable. This substitution in Group C was increased evenly at the middle of the defect, as well as at the DPB ends at 12 weeks, and the DPB remained noticeable at 12 weeks (Fig. 7).

**Fig. 8:**
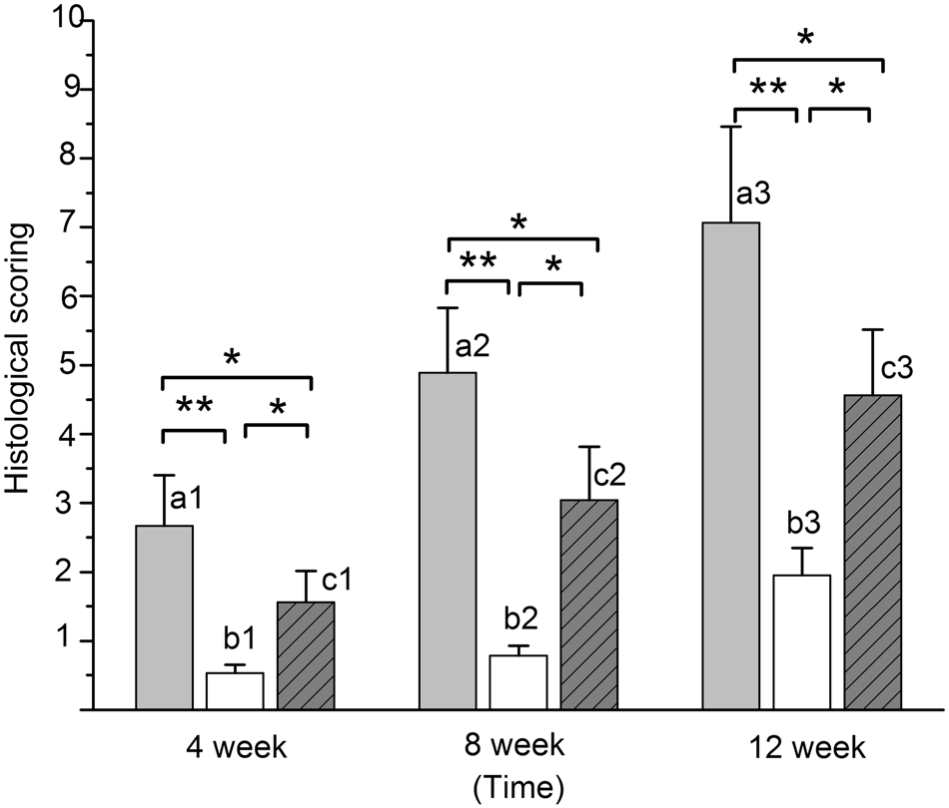
Histograms of histological scores between each group at 4, 8, and 12 weeks. The letters a1, b1, and c1 represent Groups A, B and C at 4 weeks, respectively. In the same way, a2, b2, and c2, and a3, b3, and c3 represent Groups A, B, and C at 8 and 12 weeks, respectively. Values are expressed as the mean ± S.D.; *n* = 12 per group. “*” and “**”represent significant differences between the different groups at the same time (*P < 0.05 and **P < 0.001)

Based on the quantitative analysis of new bone formation by histological inspection, the experimental groups (Group A and C) were significantly superior to the control groups (Group B) at 4, 8 and 12 weeks (*P* < 0.05 between Group B and Group C, *P* < 0.001 between Group B and Group A). The histological score of new bone formation in Group A was significantly higher than that of Group C (*P* < 0.05) at 4, 8, and 12 weeks (Fig. 8). Samples from group D were not tested because the defects failed to exhibit bone formation based on gross view assessments and radiographic inspection.

## Discussion

Critical sized defects (CSD) created in animal models are commonly used in preclinical studies to simulate large bone defects and evaluate therapeutic effects. A CSD is defined as “the smallest size bone defect on a particular bone and animal species that will not heal spontaneously during the study span” [25, 26]. Usually, a 15 mm defect is recommended as a standard CSD of a rabbit’s radius [27, 28]. In our previous studies, we observed complete bony healing of 15 mm CSDs in a NZWR model using the TEP. In the present study, we lengthened the defect to almost the complete radial shaft (averaging 40 mm, which is far beyond the CSD), to imitate bone defects of any size in clinical settings.

In this study, rabbit’s radii were selected to establish the large long bone defect model for the following reasons: (1) the radius is smaller than the ulna, it bears a minor load, and it is easily accessible [29]. (2) forearm stability and load bearing would be maintained by ulna through the interosseous membrane and ligaments after radial shaft resection. Therefore, it would not be necessary to introduce an additional internal fixation, which would increase trauma and introduce variability.

The results of this study of large size bone defects indicated that the TEP (Group A and C) construct was superior to pure allografts (Group B), and the SIS membrane (Group D) (*P* < 0.05) could restore bone defects of any length, which is meaningful for clinical situations of pathological defects.

The periosteum plays a key role in both bone development and bone healing [9, 10]. The natural periosteum is composed of extracellular matrix and periosteal stem cells that are similar in differentiation and proliferation capacity to MSCs, osteogenic progenitors, and osteoblasts [30, 31]. In the present study, as well as our previous studies, we have fabricated a very simple BTE graft with a simple architecture (2D), and the less exogenous components (Binary exogenous elements) compared to multiple exogenous elements (“triangular concept” or “diamond concept”) [32]. This simple fabrication could result in powerful in vivo osteogenesis, depending on a favorable microenvironment. Methodologically, we implanted TEP intentionally using principle of in vivo reactor (IVB) to form a relatively closed chamber. The principle of IVB focuses on initializing the body’s own regenerative capacity to regenerate new tissue, and using the body as a bioreactor to cultivate traditional implants [1, 33]. In the process of surgical implantation, we enveloped the large defect gap with the TEP or SIS membrane using a specific suturing method to create an IVB. The SIS group did not generate any new bone in such an artificial space, whereas the TEP group formed substantial new bone and attained a complete bony union. These differing results implied that the TEP membranous implant could create an osteogenic compartment using the closed suturing method, and regenerate new bone by simulating an IVB. Based on the results, we speculate that seeding cells might be an essential element to initiating the IVB for bone regeneration.

As is known, MSCs are a fundamental element of bioengineered bone constructs. Many studies have demonstrated that the in vivo osteogenic activity of constructs is mainly produced by MSCs [33]. Moreover, MSCs can secrete osteo-inductive growth factors, which may direct more host bone-forming cells toward osteogenesis when implanted in vivo [30, 34, 35].

Stevens et al. reported that an osteogenic IVB created between the tibia and the periosteum in a NZWR model restored bone defects [36]. However, the model was not consistent with the clinical situation in which pathological defects caused by trauma or disease are usually accompanied by a damaged/absent periosteum and damaged soft tissue [37–39]. Our study demonstrated that implant of BTE was not necessarily fabricated in vitro with too much exogenous elements. Using the IVB principle, a simple implant could also regenerate bone defects of any size.

## Conclusions

TEP combined with the principle of IVB successfully restored defects of all sizes in allogeneic rabbits, whereas SIS, and the pure scaffold with free cells, did not result in bone healing. Moreover, newly formed bone via TEP and the IVB method could regenerate mature long bones within 12 weeks in the rabbit model, without inducing immune reactions. Thus, such methods provide alternative approaches to restoring pathological bone defects in clinical settings.

## Abbreviations

BTE: Bone tissue engineering
3D: Threee-dimentional
IVB: in vivo bioreactor
TEP: Tissue-engineered periosteum
MSCs: Mesenchymal stem cells
SIS: Small intestinal submucosa
CSD: Critical size defect
NZWRs: New Zealand white rabbits
DMEM: Dulbecco’s modified Eagle medium
FBS: Fatal bovine serum
CO2: Carbon dioxide
EDTA: Ethylenediaminetetraacetic acid
ALP: Alkaline phosphatase activities
PBS: Phosphate buffered solution
SDS: Sodium dodecyl sulphate;
Co-60: Cobalt-60
kGy: Kilogray
DPB: Deproteinized bone;
SEM: Scanning electron microscopy
HE: Hematoxylin & eosin
ROI: Region of interest
M ± SD: Mean ± standard deviation
ANOVA: One-way analysis of variance
